# Myelodysplastic Syndrome Presenting as Amegakaryocytic Thrombocytopenia in a Collodion Baby

**DOI:** 10.1177/2324709615605637

**Published:** 2015-09-10

**Authors:** Mohammed Al Pakra, Abdullah Al Jabri, Ehab Hanafy

**Affiliations:** 1 King Salman Armed Forces Hospital, Tabuk, Kingdom of Saudi Arabia

**Keywords:** amegakaryocytic thrombocytopenia, CAMT, collodion baby, ichthyosis, myelodysplasia, MDS

## Abstract

We report a rare case of myelodysplastic syndrome that presented early as amegakaryocytic thrombocytopenia in a collodion baby, which is a rare congenital disorder characterized by thick, taut membrane resembling oiled parchment or collodion, which is subsequently shed. To our knowledge, this is the first reported case of a collodion baby who presented with amegakaryocytic thrombocytopenia and who has a significant family history of the same condition. We document the rarity of this possible association and also the need for further study to establish whether a causal relationship exists.

## Introduction

Amegakaryocytic thrombocytopenia is a hematological disorder characterized by severe thrombocytopenia, probably due to an immunologically induced absence of megakaryocytes in an otherwise normal appearing bone marrow.^1^ It is a rare condition that could be associated with other diseases. However, in our case we are presenting another rare association with a baby having a skin disorder and significant family history of same disease among his siblings. The child’s skin condition improved with time but the thrombocytopenia persists, and he developed full picture of myelodysplastic syndrome (MDS), which was detected during follow-up bone marrow aspirates, and the condition still needs extensive supportive measures to maintain the child’s quality of life.

## Case

We report the case of a 6-year-old, full-term male infant delivered by emergency caesarian section due to fetal bradycardia, birth weight of 2.3 kg, and who was admitted at the neonatal intensive care unit requiring intubation, ventilation, and treatment of methicillin-resistant *Staphylococcus aureus* sepsis. He was diagnosed clinically as a collodion baby since birth, which was confirmed later with a skin biopsy.

The child has significant family history as his parents are first cousins, with history of previous 10 miscarriages and previous 3 siblings dying at ages of 2 weeks, 1 month, and 1½ years, respectively, having the same genetic skin disorder and severe thrombocytopenia.

On initial examination, he was alert, conscious, not pale or jaundiced, not dysmorphic, with flat anterior fontanel, slightly blue sclera, and bilateral ectropion of the eye lids. There was generalized dry skin, covered with scaly crusted lesions more over the hands, feet, and back. There were eroded fingers and toes with abnormal contractures. Other systems review was unremarkable.

Incidentally, during routine investigations he was found to have persistent thrombocytopenia with platelet count ranging from 5000 to 20 000, while other complete blood count parameters were within normal values.

Bone marrow aspirate showed adequate cellularity, slightly reduced erythropoiesis, granulocytes were normal in shape and distribution, markedly reduced megakaryocytes with only one immature atypical megakaryocyte, and the picture was highly suggestive of congenital amegakaryocytic thrombocytopenia.

The patient was diagnosed as having congenital amegakaryocytic thrombocytopenia and started regular follow-up at the hematology clinic.

He presented in subsequent visits with signs of bleeding (petechial hemorrhage, ecchymosis, epistaxis, etc), and platelet transfusion was reserved for active bleeding only.

Unfortunately the child has no HLA-matched donor for bone marrow transplantation, which offers the only possibility of cure in these patients; moreover, the skin condition would have interfered with such an aggressive treatment if a matched donor was available.

Later on the child had progressive disease, when he developed huge hepatosplenomegaly and blood counts showed pancytopenia, and at this stage another bone marrow aspirate was done and showed a picture of MDS. After that, the child was kept under follow-up at the hematology clinic, which offering him supportive measures in a palliative intent and blood products in case of evident bleeding or severe anemia.

## Discussion

Congenital amegakaryocytic thrombocytopenia (CAMT) is a rare disorder expressed in infancy and characterized by isolated thrombocytopenia and megakaryocytopenia with no physical anomalies. Muraoka and colleagues reported that a patient with CAMT had a defective response to thrombopoietin (TPO) in megakaryocyte-colony formation and decreased numbers of erythroid and myelocytic progenitors in clonal cultures.^2^

The TPO receptor is defective due to mutations in the c-MPL gene.^3^ TPO binding stimulates both early and late phases of megakaryocytopoiesis, increasing the number, size, and ploidy of megakaryocytes and promoting the expression of platelet-specific markers.

However, TPO has no or little effect on platelet shedding from megakaryocytes.^4^ TPO is not only the most important growth factor for megakaryocytopoiesis, but it is also involved in maintaining the numbers of hematopoietic stem cells; this fact contributing to explain the occurrence of both thrombocytopenia and pancytopenia in CAMT patients.

Interestingly, different types of mutations have been associated with different phenotypes, allowing patients to be subdivided into 2 groups. Mutations predicted to result in a complete loss of function of the TPO receptor led to more severe thrombocytopenia and early onset of pancytopenia, whereas missense mutations were associated with transient increases of platelet counts during the first year of life and late or no development of pancytopenia.^5^

Conventional treatment modalities, like corticosteroids, androgens, immunosuppressive drugs, or splenectomy, have been ineffective. In vitro studies revealed that thrombocytopenia in CAMT cannot be corrected by administration of TPO.^2^ Platelet transfusion should be used discretely. Platelet numbers should not be the sole indication; clinical bleeding is the more appropriate trigger for the use of platelets. Moreover, it should be used cautiously to avoid refractory alloimmunization.

Bone marrow transplantation (BMT) offers the only possibility of cure for these patients. There are few reports of BMT in CAMT patients, but some of them showed difficulty in achieving engraftment and a more aggressive preparative regimen or an increased stem cell dose was suggested to be necessary to achieve engraftment.^6-8^ The results of allogeneic BMT are better in untransfused children so it is recommended to be done prior to the development of aplastic anemia.^6,9^

Recent studies have shown that peripheral blood stem cell transplantation results in rapid hematological and immunological recovery without excessive acute graft-versus-host disease compared with BMT. This contributes to less transfusion before hematological recovery, early discharge, and lower transplantation costs.

Amegakaryocytic thrombocytopenia may be a primary disorder itself^10^ or may be seen in aplastic anemia,^11^ preleukemia,^11^ and in systemic lupus erythematosus.^12-14^ It has also been reported in patients with Graves’ disease treated with radioiodine in the past,^15^ in congenital rubella, dengue fever, nutritional B_12_ deficiency, ethanol abuse, and certain congenital disorders like the thrombocytopenia-absent radius (TAR) syndrome.

Progression of amegakaryocytic thrombocytopenia to aplastic anemia or MDS/acute myelogenous leukemia is suggestive of a defective common progenitor derived from pluripotent or early committed stem cell as a result of bone marrow microenvironment injury.^16^

In our study, we report a rare association of amegakaryocytic thrombocytopenia that progressed to MDS in a collodion baby with significant family history of the same condition.

On the other hand, a collodion baby is covered at birth by a thick, taut membrane resembling oiled parchment or collodion, which is subsequently shed.

The condition is usually a manifestation of congenital ichthyosiform erythroderma or lamellar ichthyosis. As with harlequin fetus, a collodion baby appears to be one phenotype for several genotypes.

Less commonly, collodion babies evolve into other forms of ichthyosis or Gaucher disease, and a small subset is otherwise healthy without chronic skin disease. Affected neonates have ectropion, flattening of the ears and nose, and fixation of the lips in an O-shaped configuration. Hair may be absent or may perforate the horny covering. The membrane cracks with initial respiratory efforts and, shortly after birth, begins to desquamate in large sheets. Complete shedding may take several weeks, and a new membrane may occasionally form in localized areas.^17-21^

Chanarin-Dorfman syndrome, also known as neutral lipid storage disease with ichthyosis, is an autosomal recessive form of nonbullous congenital ichthyosiform eryhthroderma. Babies with Chanarin-Dorfman may be born with a collodion membrane and affected individuals have other organ involvement, the most frequent of which is hepatomegaly and liver steatosis. However, this syndrome was not documented to be associated with CAMT or MDS.^22,23^

Neonatal morbidity and deaths may be due to cutaneous infection, aspiration pneumonia, hypothermia, or hypernatremic dehydration from excessive transcutaneous fluid losses as a result of increased skin permeability. The outcome is uncertain, and accurate prognosis is impossible with respect to the subsequent development of ichthyosis.

In conclusion, we emphasize the rarity of this possible association, and also the need for further molecular and genetic studies to establish whether a causal relationship exists.
